# Molecular Assessment of Genes Linked to Honeybee Health Fed with Different Diets in Nuclear Colonies

**DOI:** 10.3390/insects16040374

**Published:** 2025-04-02

**Authors:** Worrel A. Diedrick, Lambert H. B. Kanga, Rachel Mallinger, Manuel Pescador, Islam Elsharkawy, Yanping Zhang

**Affiliations:** 1 Entomology Department, College of Agriculture and Food Sciences, Florida A&M University, Tallahassee, FL 32307, USA; worrel1.diedrick@famu.edu (W.A.D.); manuel.pescador@gmail.com (M.P.); 2 Department of Entomology and Nematology, University of Florida, Gainesville, FL 32608, USA; rachel.mallinger@ufl.edu; 3 Center for Viticulture and Small Fruits Research, Tallahassee, FL 32307, USA; islam.elsharkawy@famu.edu; 4 Interdisciplinary Center for Biotechnology Research, University of Florida, Gainesville, FL 32610, USA; yanp@ufl.edu

**Keywords:** honeybee colonies, gene expression, immune response, monofloral and polyfloral diets, pollen patty, sugar syrup, honeybee health, Varroa mite, companion crops, landscape

## Abstract

Honeybees are of economic importance not only for honey production, but also for crop pollination, which amounts to USD 20 billion per year in the United States. However, the number of honeybee colonies has declined more than 40% during the last few decades. Although this decline is attributed to a combination of factors (parasites, diseases, pesticides, and nutrition), unlike other factors, the effect of nutrition on honeybee health is not well documented. In this study, we assessed the differential expression of seven genes linked to honeybee health under three different diets. These included immune function genes [*Cactus*, immune deficiency (*IMD*), *Spaetzle*)], genes involved in nutrition, cellular defense, longevity, and behavior (*Vitellogenin*, *Malvolio*), a gene involved in energy metabolism (*Maltase*), and a gene associated with locomotory behavior (*Single-minded*). The diets included (a) commercial pollen patties and sugar syrup, (b) monofloral (anise hyssop), and (c) polyfloral (marigold, anise hyssop, sweet alyssum, and basil). Over the 2.7-month experimental periods, adult bees in controls fed pollen patties and sugar syrup showed upregulated *Cactus* (involved in Toll pathway) and *IMD* (signaling pathway controls antibacterial defense) expression, while their counterparts fed monofloral and polyfloral diets downregulated the expression of these genes. Unlike *Cactus* and *IMD*, the gene expression profile of *Spaetzle* (involved in Toll pathway) did not differ across treatments during the experimental period except that it was significantly downregulated on day 63 and day 84 in bees fed polyfloral diets. The *Vitellogenin* gene indicated that monofloral and polyfloral diets significantly upregulated this gene and enhanced lifespan, foraging behavior, and immunity in adult bees fed with monofloral diets. The expression of *Malvolio* (involved in sucrose responsiveness and foraging behavior) was upregulated when food reserves (pollen and nectar) were limited in adult bees fed polyfloral diets. Adult bees fed with monofloral diets significantly upregulated the expression of *Maltase* (involved in energy metabolisms) compared to their counterparts in control diets to the end of the experimental period. *Single-Minded Homolog* 2 (involved in locomotory behavior) was also upregulated in adult bees fed pollen patties and sugar syrup compared to their counterparts fed monofloral and polyfloral diets. Thus, the food source significantly affected honeybee health and triggered an up- and downregulation of these genes, which correlated with the health and activities of the honeybee colonies. Overall, we found that the companion crops (monofloral and polyfloral) provided higher nutritional benefits to enhance honeybee health than the pollen patty and sugar syrup used currently by beekeepers. Furthermore, while it has been reported that bees require pollen from diverse sources to maintain a healthy physiology and hive, our data on nuclear colonies indicated that a single-species diet (such as anise hyssop) is nutritionally adequate and better or comparable to polyfloral diets. To the best of our knowledge, this is the first report indicating better nutritional benefits from monofloral diets (anise hyssop) over polyfloral diets for honeybee colonies (nucs) in semi-large-scale experimental runs. Thus, we recommend that the landscape of any apiary include highly nutritious food sources, such as anise hyssop, throughout the season to enhance honeybee health.

## Simple Summary

Agriculture increasingly depends on honeybees for crop pollination, and the added value of crops pollinated by honeybees has been estimated at USD 20 billion; it is, therefore, vital to sustaining a profitable agriculture industry. Unfortunately, the number of colonies has continued to decline over the last few decades. Colony losses have occurred concurrently with an increasing demand for the pollination of fiber, fruit, vegetable, and nut crops. This decline is attributed to a combination of factors including parasites, diseases, pesticides, and nutrition. In this study, using nuclear colonies (nucs), we assessed the impact of different diets on the expression of genes linked to honeybee health. These included immune function genes [*Cactus*, immune deficiency (*IMD*), *Spaetzle*)], genes involved in nutrition, cellular defense, longevity, and behavior (*Vitellogenin*, *Malvolio*), a gene involved in energy metabolism (*Maltase*), and a gene associated with locomotory behavior (*Single-minded*). The diets included (a) commercial pollen patties and sugar syrup, (b) monofloral (anise hyssop), and (c) polyfloral (marigold, anise hyssop, sweet alyssum, and basil). At the end of a 2.7-month experimental period, we found that the diets significantly affected honeybee health and triggered an up- and downregulation of these genes, which correlated with the health and activities of the honeybee colonies. Overall, we found that monofloral and polyfloral diets provided higher nutritional benefits, thus enhancing honeybee health, compared with the pollen patty and sugar syrup. Unlike the findings in several reports on honeybee nutrition, our data indicated that a single-species diet (such as anise hyssop) is nutritionally adequate and better or comparable to polyfloral diets. However, this result will depend on the nutritional value and bloom period of individual plant species. Thus, we recommend that the landscape of any apiaries include resource-rich and abundant flowering plants, like anise hyssop, throughout the season to enhance honeybee health.

## 1. Introduction

Food production and forest ecosystems are largely dependent on insect pollinators. The implications of pollinator health (location, density, and activity) are important, with a direct economic value worldwide greater than USD 175 billion [1,2,3]. Unfortunately, bees and other insect pollinators have suffered a sharp population decline in recent decades caused by parasites, pesticides, pathogens, land development, and habitat fragmentation [4,5,6]. The detrimental effects of pesticides, parasites, and pathogens on honeybee health have been well documented [7,8,9,10]. Honeybees have an innate immune system, which include cellular and humoral responses against pathogens and parasites [11]. Cellular responses consist of phagocytosis, encapsulation, and myelinization mechanism [12]. The humoral response system includes pattern recognition receptors that interacts with pathogen-associated molecules which stimulate different pathways (Toll, IMD, JNK, JAK/STAT) depending on each type of pathogen to produce anti-microbial peptides (AMPs) [13]. The immune system of individual bees plays a key role in colony health status [13,14]. For example, using relish (IMD pathway) and defensin (Toll pathway) as immune markers is useful for monitoring colony health when compared to using of deformed wing virus or mite load for assessing colony health [15]. Honeybees are generalist pollinators, having relationships with several plant species for their nutritional requirements, to ensure the normal growth and development of bee colonies [16,17]. Floral species produce pollens which differ in their nutritional contents; the pollen quality and availability of polyfloral pollen diets influence pollen intake, which, in turn affect, honeybee health. Some immune functions, particularly glucose oxidase (GOX) activity (an enzyme involved in the synthesis of antiseptic hydrogen peroxide in honey), can be enhanced by polyfloral diets when compared with monofloral diets [18]. Overall, the health of honeybee colonies is maintained not by a monospecific pollen diet, but by a more diverse pollen diet which augments the lifespan of honeybees [18]. Moreover, worker bees fed with a polyfloral pollen diet can have increased immune functions against pathogenic invasions [19]. It has been shown that honeybees fed with a diverse pollen diet had a higher level of glucose oxidase and brood food compared with those fed with a monofloral diet with a higher protein content [19]. The pollen preferences of the foragers are determined by the requirements of the colony; preferred pollen sources are influenced more by the composition of fatty and amino acids of the pollen than by the total protein content [16,17]. Pollen and nectar are essential dietary sources for honeybees [20]. Pollen contains proteins, amino acids, lipids, vitamins, and minerals, all of which are vital for honeybee health, colony growth and development [20,21]. The number and health of honeybees in the landscapes can be increased through habitat restoration and other land management practices [21]. Little is known about factors related to nutritional fitness and the composition of the landscape on honeybee health. Previous studies suggested that strategies should include a mixture of plant species (companion crops) to provide continual blooms and complementary nutritional profiles throughout the growing season for use by insect pollinators. Indeed, reduced floral resource diversity can lead to suboptimal bee nutrition, resulting in a weakened immune system and poor overall bee health [22,23]. However, in some cases, highly nutritious plants with a long bloom period may be adequate for generalist pollinators, like honeybees [24].

Diet is an important factor in the development of individuals and the entire colony. Alaux et al. [19] reported that an improperly balanced diet may weaken the immune system and increase the susceptibility of workers to diseases. Bryś et al. [25] reported that a honeybee diet based on multiflower pollen is more desirable than a monoflower diet but must be properly balanced. In addition, while pollen from a single species has specific benefits, bees may require pollen from diverse sources to maintain their individual health and a healthy hive.

The objectives of this study were to assess the impact of nutrition on honeybee health. These include (a) the strength and growth of the honeybee colony, and (b) the response of genes linked to bee health when provided with different diets. We compared the differential expression of seven genes, including immune function genes (*Cactus*, immune deficiency, *Spaetzle),* genes involved in nutrition, longevity, and behavior (*Vitellogenin*, *Malvolio*), a gene involved in energy metabolism (*Maltase*), and a gene associated with locomotory behavior (*Single-minded*) linked to honeybee health in adult bees when fed with the following three diets: a control diet (commercial pollen and sugar patties), a monofloral diet (anise hyssop), and a polyfloral diet (companion crops). To the best of our knowledge, this is the first study of its kind using nuclear (nuc) honeybee colonies. The findings should provide useful insights into the development of the best beekeeping practices.

## 2. Materials and Methods

### 2.1. Honeybee Colonies and Treatments

**Figure 1:**
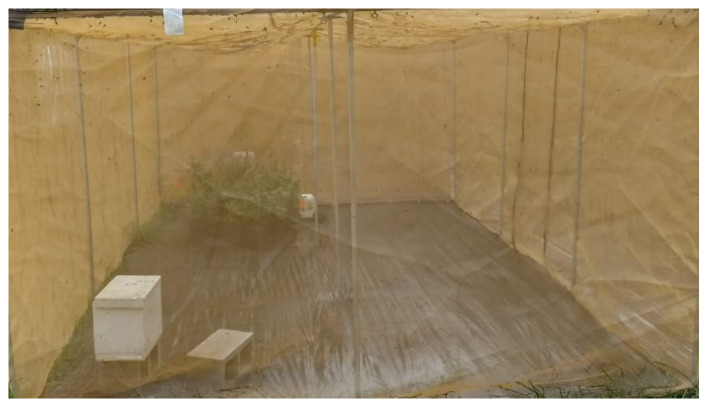
Flight cage with nuclear honeybee colony, companion plants, and water source.

**Figure 2:**
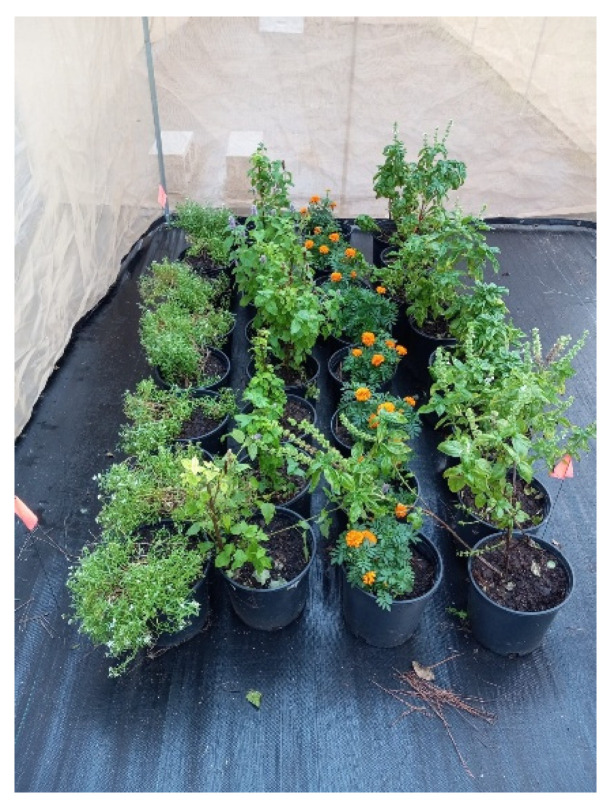
Companion plants (sweet alyssum, anise hyssop, marigold, and basil) inside the flight cage.

Twenty-one colonies with uniform honeybee (*Apis mellifera*) populations were established in our apiary at the Research Experimental Farm in Quincy, FL. The experiments were conducted in 15 flight cages (Figure 1) (each 6 m × 3 m × 1.8 m, or 32.4 m^2^), with 5 flight cages per treatment and 1 nuclear (nuc) colony per flight cage, totaling 15 colonies. The honeybees were provided with three diets (treatments). Treatment 1 (control) received commercial pollen patties (Dadant & Sons, Inc., Hamilton, IL, USA) and sugar syrup. Treatment 2 (monofloral) received anise hyssop (*Agastache foeniculum*). Treatment 3 (polyfloral) was provided with a companion crop mix [anise hyssop (*A. foeniculum*), marigold (*Calendula officinalis*), sweet alyssum (*Lobularia maritima*) and basil (*Ocimum basilicum*)]. The companion crops were evenly spaced in the flight cages and covered 10% of the total area to serve as a food source (pollen, nectar) for the bees [26]. A water source was also provided to the bees (Figure 1). The companion plants were chosen because of their high nutritional benefits (nectar and pollen), long blooming period, attractiveness to honeybees, and easiness to grow [27,28,29,30]. Honeybees also select plants to forage based on their colony’s needs [31]. The food sources (companion plants) made up 10% of the total area [26] (Figure 1 and Figure 2). The experiments ran from 4 October to 27 December 2023.

### 2.2. Assessment of Varroa Mite Infestations and Honeybee Colony Strength

Mite infestations of adult bees in the nucs were assessed at the start of the experiments and at 84 days post-treatment using the alcohol wash method as described by Delaplane et al. [32]. The strength of the bee colonies was evaluated by visually inspecting both sides of each frame in the hive to estimate the area covered with adult bees, brood, pollen, and honey. The estimates were made in tenths of frame equivalents (for a standard 232 mm-deep Langstroth frame [33,34]. The assessments of colony strength were conducted at the beginning and at the end of the 84-day experimental period.

### 2.3. Honeybee Samples

Samples of 9 adult female worker bees were collected from each of the 5 treatments every 21 days. Adult bees were randomly collected from the frames and placed in plastic crush-proof containers. Samples were held on liquid nitrogen before being stored in a −80 °C freezer. Samples were later processed using reverse transcription droplet digital PCR (RT-ddPCR).

### 2.4. RNA Extraction and Reverse Transcription

Adult honeybees were ground in liquid nitrogen. Ribonucleic acid (RNA) was isolated from nine individual worker bees using the RNeasy Mini Kit (Qiagen, Hilden, Germany, catlog #74104) according to the manufacturer’s protocol. RNA concentration was quantified using the Qubit^®^ 2.0 Fluorometer method (Invitrogen, Waltham, MA, USA) and RNA quality was assessed using the Agilent TapeStation 2200 (Agilent Technologies, Inc., Santa Clara, CA, USA). Then, 500 ng of total RNA was used for reverse transcription using SuperScript™ IV VILO™ Master Mix (Thermo Fisher, Waltham, MA, USA, Cat. No: 11766050) according to the user guide.

### 2.5. Primer Design

The potential impact on the function of genes linked to honeybee health were assessed from a subset of known genes (Table 1) from the literature [35,36,37]. These include genes involved in nutrition and cellular defense [*Cactus*, immune deficiency (*IMD*), *Spaetzle* (Spz), *Malvolio* (Mvl), and *Vitellogenin* (Vg)] and genes involved in energy and behavior (*Maltase* and *Single-minded homolog* 2). Primers were designed using information from the National Center for Biotechnology Information, (http://www.ncbi.nlm.nih.gov, NCBI) and Bee Base (http://hymenopteragenome.org). Primer conditions were optimized by determining the optimal annealing temperature (Ta) and primer concentration [38]. Before real-time PCR (RT-PCR), each primer was validated by specific PCR amplifications and 1.5% agarose gel electrophoresis in TBE buffer with ethidium bromide staining.

### 2.6. Droplet Digital Polymerase Chain Reaction (ddPCR)

Droplet digital PCR amplification was performed with nine individual adult bees collected from each of the five treatments. The oligonucleotide amplification primers are listed in Table 1. The gene expressions of *Cactus*, immune deficiency, *Malvolio*, *Maltase*, *Single*-*Minded*, Spaetzle, and *Vitellogenin* were tested using a BioRad QX200 AutoDG Droplet Digital PCR System (Bio-Rad, Hercules, CA, USA). Briefly, each of the 20 μL reactions contained 10 uL QX200 EvaGreen Supermix (Bio-Rad, Hercules, CA, USA), 250 nM gene-specific primers, and 1 μL of the cDNA sample with 6.67 ng of total RNA input. The 20 µL reaction was mixed with 20 µL of droplet generation oil and partitioned in ~20,000 droplets after droplet generation. Then, the nanoliter-sized droplets were PCR amplified in a thermal cycler with enzyme activation at 95 °C for 10 min followed by a two-step cycling protocol for 40 cycles (94 °C for 30 s; 60 °C for 1 min); after enzyme deactivation at 98 °C for 10 min, the final step was to hold at 4 °C indefinitely. A ramp rate of 2 °C/s and a lid temperature of 105 °C were used. Finally, the fluorescence intensity of individual droplets was measured with the QX200 Droplet Reader (Bio-Rad). The data analysis was performed with Quantasoft droplet reader software (v. 1.4) (Bio-Rad). Positive and negative droplet populations were detected automatically. The absolute transcript expression was reported in copies/ng of total RNA input. The RNA extraction, RT, and ddPCR were performed at the University of Florida’s ICBR Gene Expression Core (https://biotech.ufl.edu/gene-expression-genotyping/ (accessed on 6 March 2023), RRID:SCR_019145). 

### 2.7. Statistical Analysis

Data on colony development and mite infestations were subjected to analysis of covariance using pre-treatment data as the covariate [39]. The data on adult bee populations, brood, pollen, and honey were transformed to log (x + 1) to satisfy the assumptions of normality before analysis. Means between treatments were separated using Tukey’s studentized range test. Data on gene expression (transcript abundances) for bee samples from the different treatments were subjected to repeated measures analysis [39]. Treatments were modeled as fixed effects; date and date-by-treatment interactions were modeled as random effects. The data on gene expression were transformed to log (x + 1) to satisfy the assumptions of normality before analysis. A significant level of alpha = 0.05 was used for all statistical tests.

## 3. Results

### 3.1. Honeybee Colony Growth and Development

The numbers of mites per adult bees were similar in all treatment groups at the start of the experiment on day 0 (Table 2). Mite infestations per adult bees did not vary significantly between treatment groups (F = 0.19; df = 2, 3; *p* = 0.8356) at the end of the 84-day experimental period. The average number of bees in the colonies did not differ between the treatments at the beginning of the experiment (day 0). At the end of the experimental run, bee populations were significantly reduced as compared to the initial size of the populations for all treatment groups (F = 8.77; df = 5, 13; *p* = 0.0008) except for the monofloral diet group (Table 2). The honey reserves were significantly different between treatment groups (F = 8.77; df = 5, 13; *p* = 0.0008) and significantly higher in adult bees fed with pollen patties and sugar syrup as compared to their counterparts fed with monofloral and polyfloral diets (Table 2). The experiments were conducted in the fall season, and the colonies (nucs) had no brood production nor pollen reserves (only traces) at the end of the 84-day experimental period. Our observations during the experimental run indicated that the pollen that was collected by adult bees was not stored but was instead probably used for the daily maintenance of the colonies.

### 3.2. Expression of Genes Linked to Honeybee Health Fed with Different Diets

#### 3.2.1. *Cactus* Gene Expression

**Figure 3:**
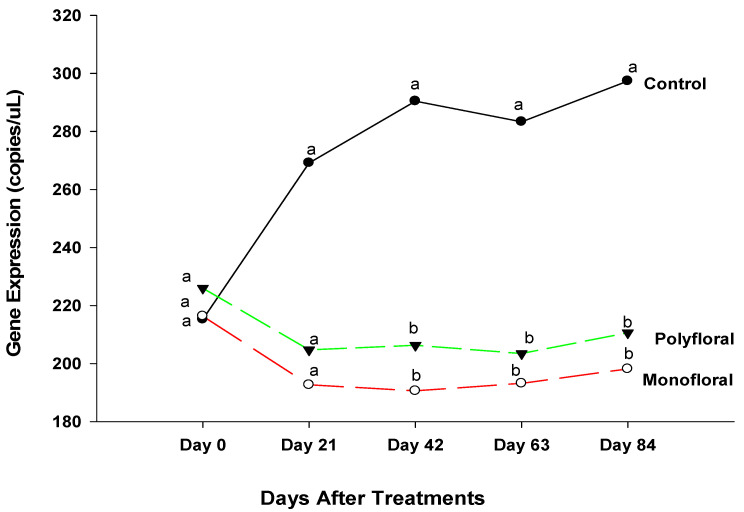
Patterns of the gene expression profile of *Cactus* in adult bees fed with control (pollen and sugar syrup), monofloral (anise hyssop), and polyfloral (companion crops) diets during the 84-day experimental period. Means between lines within each sampling date are not significantly different if followed by the same lower-case letter (*p* > 0.05; Tukey test).

Expression levels (transcript abundances) of *Cactus*, a gene involved in the immune response in the Toll pathway [40,41] were similar in all treatment groups in flight cages at the start of the experiments on day 0 (F = 0.05; df = 2, 3; *p* = 0.9476) and on day 21 (F = 0.47; df = 2, 3; *p* = 0.6658) (Figure 3). However, on day 42, gene expression varied significantly between control groups fed with pollen patties and sugar syrup and their counterparts fed with the monofloral (anise hyssop) and polyfloral (companion crops) diets (F = 9.46; df = 2, 6; *p* = 0.0140). Gene expression levels of *Cactus* on day 42 were similar (t = −0.36; df = 3; *p* = 0.7445) in adult bees fed with monofloral and polyfloral diets. On day 63, differential gene expression levels of adult bees were significantly different between the control groups and both monofloral and polyfloral groups (F = 10.89; df = 2, 9; *p* = 0.0040) but not between the monofloral and polyfloral diets (t = −0.28; df = 3; *p* = 0.7958). At the end of the 84-day experimental period, gene expression levels varied significantly between treatments (F = 15.19; df = 2, 12; *p* = 0.0005). The time effect (a measure of within-treatment variability over time) was not significant (F = 1.79; df = 4,12; *p* = 0.1949) nor were the time-by-treatment interactions (F = 2.04; df = 8,12; *p* = 0.1280). However, gene expression levels of *Cactus* did not differ (t = −0.30; df = 3; *p* = 0.7832) between the monofloral and polyfloral diets on day 84. Overall, adult honeybees in the control groups fed with pollen patties and sugar syrup upregulated *Cactus* gene expression compared to their counterparts fed with monofloral (anise hyssop) and polyfloral (companion crops) diets (Figure 1).

#### 3.2.2. Immune Deficiency Gene Expression

**Figure 4:**
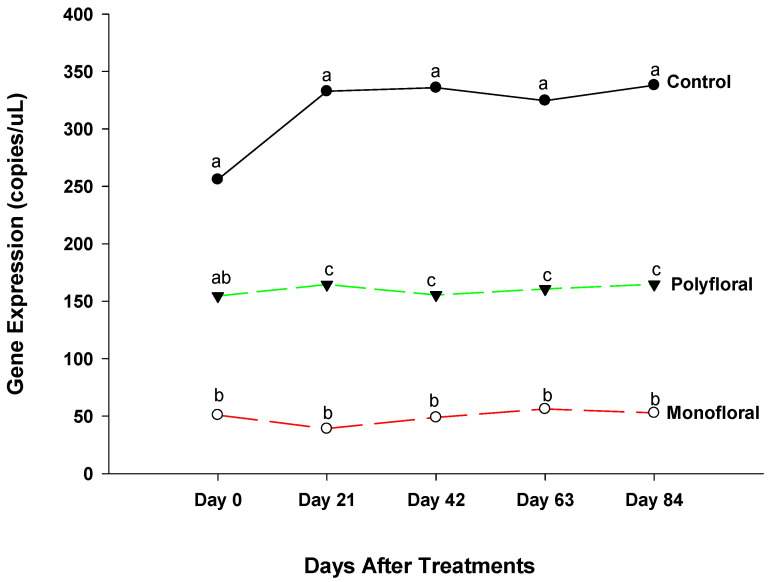
Patterns of the gene expression profile of immune deficiency in adult bees fed with control. (pollen and sugar syrup), monofloral (anise hyssop), and polyfloral (companion crops) diets during the 84-day experimental period. Means between lines within each sampling date are not significantly different if followed by the same lower-case letter (*p* > 0.05; Tukey test).

The gene expression levels of immune deficiency (*IMD*) (an immune defense pathway against bacteria and fungi) [42] were different (F = 20.27; df = 2, 3; *p* = 0.0181) between treatments at the start of the experiments on day 0 (Figure 4). Gene expression continued to vary significantly between treatments on day 21 (F = 188.15; df = 2, 3; *p* = 0.0007). Thus, adult bees fed with pollen patties and sugar syrup displayed significantly higher expression of the *IMD* gene than their counterparts fed with monofloral (anise hyssop) diets (t = 19.33; df = 3; *p* = 0.0003) and polyfloral diets (t = 11.07; df = 3; *p* = 0.0016). Also, adult bees fed with monofloral diets had a significantly lower expression of *IMD* gene compared to their counterparts fed with polyfloral diets (t = −8.26; df = 3; *p* = 0.0037). Differential gene expression levels of adult bees were significantly different between the control groups and both monofloral and polyfloral groups on day 42 (F = 88.57; df = 2, 3; *p* = 0.0021) and day 63 (F = 51.90; df = 2, 3; *p* = 0.0047). The patterns of the *IMD* gene profile for days 42 and 63 were similar to that of day 21. At the end of the 84-day experimental period, gene expression levels of *IMD* gene varied significantly between treatments (F = 65.98; df = 2, 3; *p* = 0.0033) as in previous sampling dates. The time effect was not significant (F = 1.96; df = 4,12; *p* = 0.1649) but the time-by treatment interactions were significant (F = 3.10; df = 8, 12; *p* = 0.0382). Adult bees fed the control diets displayed a significantly upregulated expression of *IMD* gene compared to those fed with monofloral diets (t = 11.40; df = 3; *p* = 0.0014) and those fed with polyfloral diets (t = 6.93; df = 3; *p* = 0.0062). Adult bees fed with monofloral diets had a significantly lower expression of *IMD* gene compared to their counterparts fed with polyfloral diets (t = −4.47; df = 3; *p* = −0.0209).

#### 3.2.3. *Spaetzle* Gene Expression

**Figure 5:**
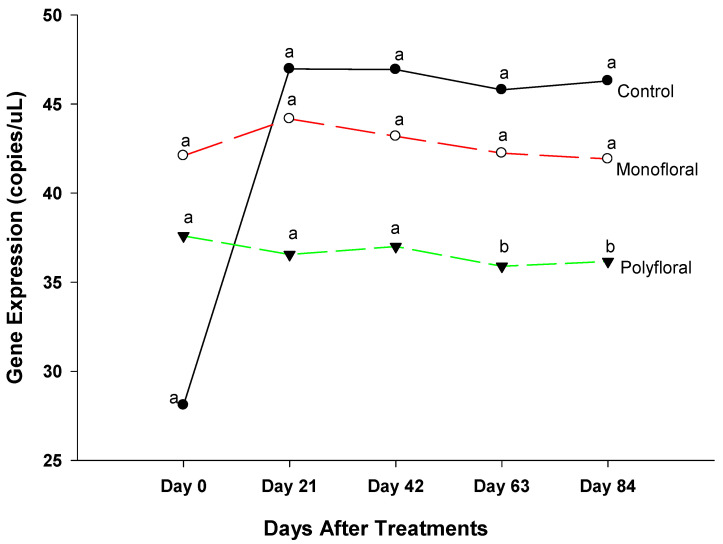
Patterns of the gene expression profile of *Spaetzle* in adult bees fed with control (pollen and sugar syrup), monofloral (anise hyssop), and polyfloral (companion crops) diets during the 84-day experimental period. Means between lines within each sampling date are not significantly different if followed by the same lower-case letter (*p* > 0.05; Tukey test).

The expression levels of the gene *Spaetzle* (Spz), a member of the Toll immune signaling pathway against fungi and bacteria [36,42], were similar (F = 2.0; df = 2, 3; *p* = 0.2805) in all treatment groups at the start of the experiments on day 0, on day 21 (F = 2.55; df = 2, 3; *p* = 0.2257), and on day 42 (F = 4.20; df = 2, 3; *p* = 0.1348) (Figure 5). However, gene expression varied significantly between treatments (F = 10.71; df = 2, 3; *p* = 0.0242) on day 63. The *Spaetzle* expression levels were similar between the control and monofloral diets (t = 0.46; df = 3; *p* = 0.6751), but not polyfloral diets (t = 4.22; df = 3; *p* = 0.0344). At the end of the experiments on day 84, the expression of Spz was significantly different (F = 8.61; df = 2, 3; *p* = 0.0501) between treatments. The time effect was not significant (F = 2.66; df = 4, 12; *p* = 0.0849) nor were the time-by-treatment interactions (F = 1.85; df = 8, 12; *p* = 0.1633). Adult honeybees in control groups fed with pollen patties and sugar syrup and in monofloral (anise hyssop) groups showed significantly upregulated gene expression compared to their counterparts fed with polyfloral diets (t = 4.00; df = 3; *p* = 0.0281), and (t = 2.97; df = 3; *p* = 0.0510), respectively, at the end of the 84-day experimental period.

#### 3.2.4. *Vitellogenin* Gene Expression

**Figure 6:**
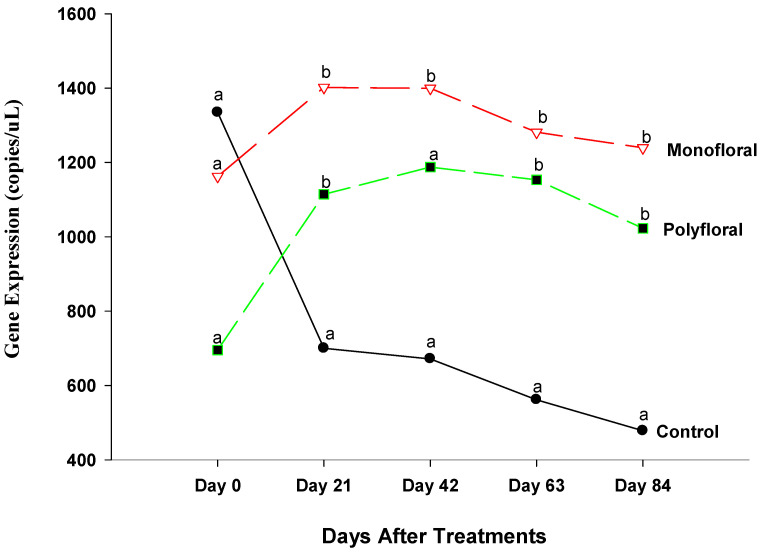
Patterns of the gene expression profile of *Vitellogenin* in adult bees fed with control (pollen and sugar syrup), monofloral (anise hyssop), and polyfloral (companion crops) diets during the 84-day experimental period. Means between lines within each sampling date are not significantly different if followed by the same lower-case letter (*p* > 0.05; Tukey test).

The expression levels of *vitellogenin* (Vg), a gene associated with immune function and longevity [36], were similar (F = 0.78; df = 2, 3; *p* = 0.5319) in all treatment groups at the start of the experiments on day 0 (Figure 6). However, differential gene expression levels of adult bees were significantly different between control groups and both monofloral and polyfloral groups on day 21 (F = 14.83; df = 2, 3; *p* = 0.0048), day 42 (F = 14.83; df = 2, 3; *p* = 0.0048), and day 63 (F = 8.71; df = 2, 3; *p* = 0.0503). Expression levels of Vg were similar between adult bees fed with monofloral and polyfloral diets on day 21 (t = 0.76; df = 3; *p* = 0.5035), day 42 (t = 0.73; df = 3; *p* = 0.5181), and day 63 (t = 0.56; df = 3; *p* = 0.6175). At the end of the 84-day experimental period, the gene expression levels of Vg varied significantly between treatments (F = 12.35; df = 2, 3; *p* = 0.0356) and followed the same pattern as on days 21, 42, and 63, as the expression levels of Vg were similar in adult bees fed with monofloral and polyfloral diets (t = 0.90; df = 3; *p* = 0.4329). The time effect was significant (F = 2.39; df = 4, 12; *p* = 0.0510), as were the time-by treatment interactions (F = 3.81; df = 8, 12; *p* = 0.0188). Adult honeybees in the control groups fed with pollen patties and sugar syrup had significantly reduced Vg gene expression compared to their counterparts fed with monofloral (anise hyssop) and polyfloral diets, and the expression levels of the gene varied during the experimental run.

#### 3.2.5. *Malvolio* Gene Expression

**Figure 7:**
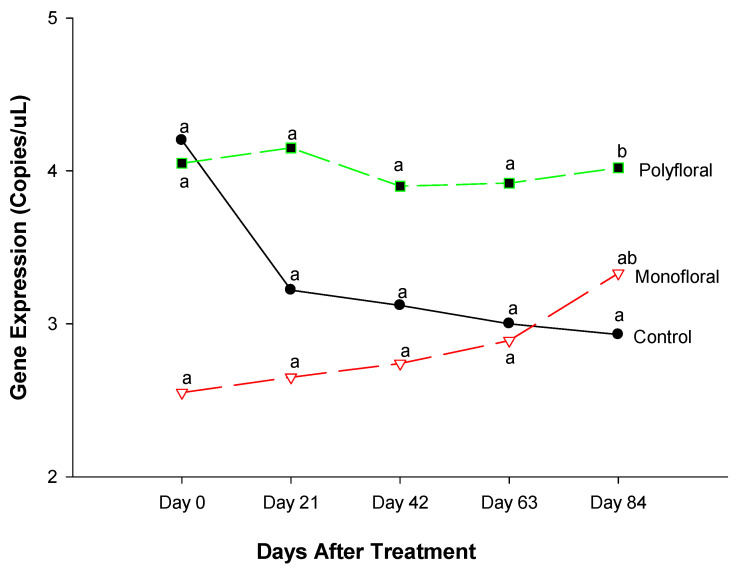
Patterns of the gene expression profile of *Malvolio* in adult bees fed with control (pollen and sugar syrup), monofloral (anise hyssop), and polyfloral (companion crops) diets during the 84-day experimental period. Means between lines within each sampling date are not significantly different if followed by the same lower-case letter (*p* > 0.05; Tukey test).

The expression level of *Malvolio* (mvl), a gene involved in sucrose responsiveness [36,43], was similar at the start of the field trials (F = 2.99; df = 2, 3; *p* = 0.1933) (Figure 7). Gene expression of mvl did not differ significantly between treatments on day 21 (F = 2.41; df = 2, 3; *p* = 0.2378), day 42 (F = 1.22; df = 2, 3; *p* = 0.4096), and day 63 (F = 0.87; df = 2, 3; *p* = 0.5022). However, the expression levels of mvl were significantly different between the treatments at the end of the experimental run on day 84 (F = 3.77; df = 2, 3; *p* = 0.0503). The time effect was not significant (F = 0.92; df = 4, 12; *p* = 0.4851), nor were the time-by-treatment interactions (F = 1.07; df = 8, 12; *p* = 0.4439). Adult honeybees fed with polyfloral diets showed upregulated mvl expression compared to their counterparts in the control groups fed with pollen patties and sugar syrup (t = 4.05; df = 3; *p* = 0.0482). Adult bees fed with monofloral (anise hyssop) diets had a similar expression of the mvl gene to that of the control groups and polyfloral groups. (t = −4.0; df = 3; *p* = 0.4647).

#### 3.2.6. *Maltase* Gene Expression

**Figure 8:**
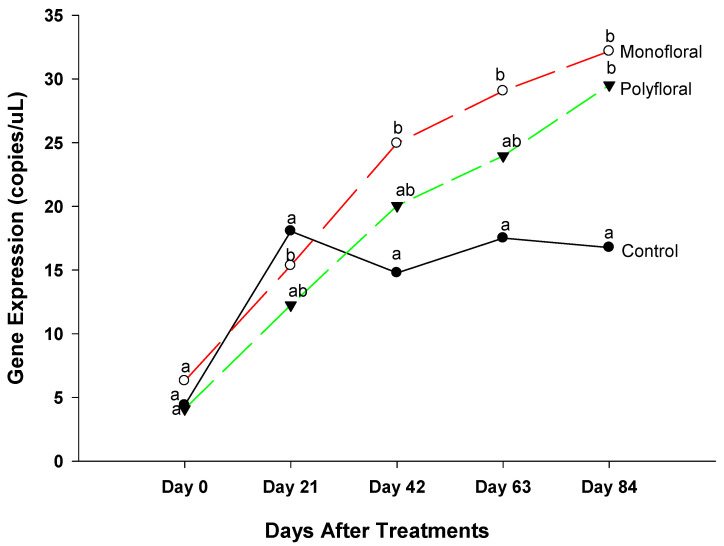
Patterns of the gene expression profile of *Maltase* in adult bees fed with control (pollen and sugar syrup), monofloral (anise hyssop), and polyfloral (companion crops) diets during the 84-day experimental period. Means between lines within each sampling date are not significantly different if followed by the same lower-case letter (*p* > 0.05; Tukey test).

The expression levels of the gene *Maltase*, a gene associated with energy metabolism [36], were similar (F = 2.73; df = 2, 3; *p* = 0.2110) in all treatment groups at the start of the experiments on day 0 (Figure 8). However, the differential gene expression levels of *Maltase* in adult bees were significantly different between the control, monofloral, and polyfloral groups on day 21 (F = 15.38; df = 2, 3; *p* = 0.0265), on day 42 (F = 14.74; df = 2, 3; *p* = 0.0281), and on day 63 (F = 9.44; df = 2, 3; *p* = 0.0501). Expression levels of *Maltase* were significantly lower in adult bees fed with pollen and sugar syrup than those of their counterparts fed with monofloral diets on day 21 (t = −5.00; df = 3; *p* = 0.0154), on day 42 (t = −3.88; df = 3; *p* = 0.0303). and day 63 (t = −3.70; df = 3; *p* = 0.0343). Adult bees in control groups had a similar expression of *Maltase* gene as their counterparts in polyfloral groups on day 21 (t = −2.34; df = 3; *p* = 0.1015), day 42 (t = −1.97; df = 3; *p* = 0.1436) and on day 63 (t = −2.07; df = 3; *p* = 0.1303). Similarly, the expression of *Maltase* did not differ between adult bees fed with monofloral and polyfloral diets on day 21 (t = 2.67; df = 3; *p* = 0.0759), on day 42 (t = 1.91; df = 3; *p* = 0.1522), and on day 63 (t = 1.62; df = 3; *p* = 0.2016). At the end of the 84-day experimental period, expression levels of *Maltase* varied significantly between treatments (F = 12.31; df = 2, 3; *p* = 0.0358). The time effect was significant (F = 84.35; df = 4,12; *p* = 0.0001) as were the time-by treatment interactions (F = 4.36; df = 8, 12; *p* = 0.0113). Adult honeybees in the control groups fed with pollen patties and sugar syrup had significantly lower expression levels of *Maltase* compared to their counterparts fed with monofloral (t = −4.74; df = 3; *p* = 0.0178) and polyfloral diets (t = −365; df = 3; *p* = 0.0355) diets. There were no significant differences (t = 1.09; df = 3; *p* = 0.3566) in gene expression levels between adult bees fed with monofloral and polyfloral diets. Overall, expression levels of *Maltase* varied over time during the 84-days of the field trials.

#### 3.2.7. *Single-Minded Homolog 2* Gene

**Figure 9:**
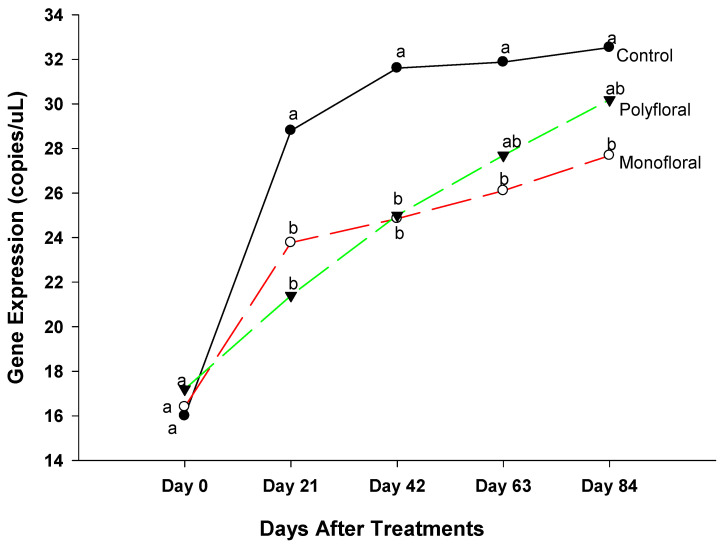
Patterns of the gene expression profile of *Single*-*minded homolog* 2 in adult bees fed with control (pollen and sugar syrup), monofloral (anise hyssop), and polyfloral (companion crops) diets during the 84-day experimental period. Means between lines within each sampling date are not significantly different if followed by the same lower-case letter (*p* > 0.05; Tukey test).

The expression levels of the gene *single*-*minded Homolog 2* (Smh2), a gene involved in locomotory behavior [36] were similar (F = 0.23; df = 2, 3; *p* = 0.8091) in all treatment groups at the start of the experiments on day 0 (Figure 9). However, the gene expression levels of adult bees were significantly different between control groups and both monofloral and polyfloral groups on day 21 (F = 41.25; df = 2, 3; *p* = 0.0066) and on day 42 (F = 55.85; df = 2, 3; *p* = 0.0001). There were no significant differences between monofloral and polyfloral diets on day 21 (t = −0.07; df = 3; *p* = 0.9467) and day 42 (t = 0.82; df = 3; *p* = 0.4722). On day 63 of the field trials, gene expression levels of *single*-*minded* were significantly different (F = 41.25; df = 2, 3; *p* = 0.0066) between the control diets and monofloral diets (t = −2.37; df = 3; *p* = 0.0507), but they were similar to that of polyfloral diets (t = 1.39; df = 3; *p* = 0.2601). At the end of the 84-day experimental period, expression levels of *single*-*minded* varied significantly between treatments (F = 4.76; df = 2, 3; *p* = 0.0300). The time effect was significant (F = 57.40; df = 4, 12; *p* = 0.0001), as were the time-by treatment interactions (F = 3.74; df = 8, 12; *p* = 0.0200). Adult honeybees in the control groups fed with pollen patties and sugar syrup had similar gene expression levels compared to their counterparts fed with polyfloral diets (t = 1.21; df = 3; *p* = 0.3142). Similarly, there were no differences (t = −1.29; df = 3; *p* = 0.2890) in gene expression between adult bees fed with monofloral and polyfloral diets. Overall, the differential gene expression of Sim2 varied over time during the 84-day experimental period.

## 4. Discussion

Our studies included immune function genes (immune deficiency, *Cactus*, and *Spaetzle*), genes involved in nutrition, cellular defense, and longevity (*Vitellogenin* and *Malvolio*), a gene involved in energy metabolism (*Maltase*), and a gene associated with locomotory behavior (*Single-minded homolog* 2). We found that the dietary treatments did not significantly affect the strength of the colonies in terms of mite infestations, but the monofloral diet group had larger colonies by the end of the experiment. Additionally, there were differences in gene expression across diets, and particularly between the monofloral and control diet. Overall, the monofloral treatment appeared to be the best diet in comparison to polyfloral and pollen and sugar patties, probably due to specific nutritional effects of anise hyssop (a companion crop).

### 4.1. Cactus Gene Expression

Adult bees fed pollen patties and sugar syrup showed upregulated *Cactus* gene expression during the 84-day experimental period while their counterparts fed with monofloral and polyfloral diets showed downregulated gene expression. The *Cactus* gene is involved in the Toll pathways, providing inflammatory responses that include the recognition of pathogens and the expression of antimicrobial peptides (AMPs) [42,43,44]. Yang and Cox-Foster [4] reported that infestations of Varroa mite contributed to stress and weakened honeybee colonies by directly affecting some genes encoding antimicrobial and immune responses. Our data indicated that Varroa mite infestation levels of adult bees were higher in the control groups (0.94 ± 0.61%) but did not differ significantly from those in the monofloral (0.41 ± 0.19%) and polyfloral groups (0.20 ± 0.12%). The mite load in our study was significantly lower than the recommended threshold (3–6 mites per 100 bees) [45] for chemical treatments. That the expression of the *Cactus* gene did not display the same trends as the Varroa mite load might suggest an absence of a strong association between mite load and the expression of the *Cactus* gene. The expression of *Cactus* was similar between the control, monofloral, and polyfloral diets up to 21 days after the experiments were initiated. Overall, the upregulation of *Cactus* in adult bees in the control groups from day 42 to the end of the experiments without any significant infestation levels of Varroa mites suggested that other factors may trigger the upregulation of the *Cactus* gene [46]. Adult bees in flight cages fed monofloral and polyfloral diets generated similar patterns of expression of this humoral defense gene during our 84-day experimental period. Whether the slightly higher Varroa mite load in control groups adversely affected the expression of *Cactus* gene is yet to be fully investigated. In a study by Barroso-Arévalo et al. [15] to assess whether the expression levels of four immune system genes (dorsal, defensin, domeless, and relish) could serve as biomarkers of colony mortality (0.3 to 28.85% mite infestation rates), they noted a decreased expression of dorsal and defensin (Toll pathway) associated with high deformed wing virus and Varroa mite loads. Maruščáková et al. [47] reported that when bees were infested with *Escherichia coli*, the up-regulation of *Cactus* led to the termination of AMP’s production. Hence, *Cactus* is a negative regulator of the Toll pathways.

### 4.2. Immune Deficiency

Expression levels of immune deficiency (*IMD*) gene in adult bees fed with pollen patties and sugar syrup in flight cage treatments were significantly upregulated throughout the 84-day experimental period compared with their counterparts fed with monofloral diets and polyfloral diets. The *IMD* signaling pathway controls antibacterial defense [48,49]. Tanji and Ip [50] and Hoffmann [51] indicated that insect antibacterial immunity relies heavily on the Toll and *IMD* pathways of the innate immune systems. Our data indicated that relatively low mite loads of 0.94 ± 61%, 0.20 ± 0.12%, and 0.41 ± 0.19% in the control, polyfloral, and monofloral groups, respectively, did not significantly change the expression of *IMD* genes within each diet from the beginning to the end of the experimental run. Although the control groups have the highest infestations of Varroa mite [(0.94 ± 0.61%) mite per 100 bees)], the rates were not within the recommended threshold (3–6 mites per 100 bees) [45] for chemical treatments. As in the Toll pathway, other factors (fungi, protozoan diseases, beekeeping practices, etc.) may trigger the upregulation of *IMD* gene expression. Our data also indicated that expression levels of *IMD* genes in adult bees fed with monofloral diets were significantly lower during the 2.7-month experimental run. In a study by Barosso-Arevalo et al. [15] on immune system genes in honeybees, he indicated that the expression of relish (*IMD* pathway) was significantly higher in honeybee colonies with high deformed wing virus and Varroa mite loads than those with low loads. Yang and Cox-Foster [4] reported that parasitic mite infestation together with deformed wing virus downregulated the expression of genes encoding antimicrobial peptides. However, the levels of Varroa mite infestations in our study might not have provided detectable effects of the expression profile of genes in the *IMD* pathway.

### 4.3. Spaetzle

We found that the expression of the *Spaetzle* gene in adult bees in controls, monofloral, and polyfloral groups was similar except on day 63 and on day 84. On these two sampling dates, adult bees fed with polyfloral diets significantly downregulated the expression of the *Spaetzle* gene. Varroa mite infestation levels of adult bees were higher in the control groups (0.94 ± 0.61%) but did not differ significantly from the monofloral groups (0.41 ± 0.19%) and polyfloral groups (0.20 ± 0.12%). In addition, this level of mite infestations was lower than the recommended threshold (3–6 mites per 100 bees) [45] for chemical treatments. Alaux et al. [7] reported that *Spaetzle* transcripts were increased in honeybees fed polyfloral pollen diets but decreased with the presence of Varroa mite. In our studies, Varroa mite load was similar between the three types of diets, suggesting that the expression of this gene was reduced, probably due to other pathways or factors (biological or environmental stressors). In our studies, *Spaetzle* expression levels in adult bees in the control diets increased significantly 21 days post-treatment, unlike in other diets, and the levels remained similar to that of monofloral diets up to the end of the 84-day experimental period. The *Spaetzle* gene is a member of the Toll immune signaling pathway against fungi and bacteria [43] that results in the production of antimicrobial peptides [52]. Overall, the expression profiles of the immune function genes (*Cactus*, immune deficiency, and *Spaetzle*) were affected by the dietary treatments (pollen and sugar patties, monofloral, and polyfloral), and adult bees fed with control diets upregulated these genes compared to their counterparts fed with monofloral and polyfloral diets.

### 4.4. Vitellogenin

Adult bees fed with monofloral and polyfloral diets displayed significantly higher expression of the *Vitellogenin* (Vg) gene compared with their counterparts in control groups fed with pollen patties and sugar syrup during the 2.7-month experimental period. Our results indicated that pollen and nectar from monofloral diets and polyfloral diets upregulated (2.7-fold) the expression of Vg. Similarly, Alaux et al. [7] and Chaimanee et al. [53] reported that expression of Vg was increased in adult bees fed with polyfloral diets. *Vitellogenin* is a major reproductive protein in insects and a proposed endocrine factor in honeybees; it has antioxidant functions that protect bees from oxidative stress and enhance longevity [54,55] affect foraging behavior, division of labor [30], and immunity [42]. Our data on colony development indicated that a significantly higher population of bees remained (longer life span) in the monofloral diet (2.92 ± 0.0.82) groups compared to their counterparts in the control diet fed with pollen patties and sugar syrup (0.72 ± 0.67) (Table 2). Reduced expression of Vg in control bees may be due to immune senescence (deterioration) in foragers due to a behavioral stimulus and/or the absence of adequate nutrients [56,57].

### 4.5. Malvolio

The expression of the Malvolio (Mvl) gene was similar between adult bees fed with control (pollen and sugar syrup), monofloral, and polyfloral diets up to day 63 post-treatment. Our data indicated that feeding adult bees with the three types of pollen and nectar did not affect Mvl expression 63 days after the experiments were initiated. However, adult bees fed polyforal diets displayed higher expression of Mvl compared to the control diets at the end of the 2.7-month experiment. The *Malvolio* gene is associated with sucrose responsiveness [58] and the division of labor in honeybees. Ben-Shahr et al. [59] and Zayed and Robinson [60] reported that foraging workers that are responsive to sucrose have higher levels of Mvl in the brain than nurse bees, and that upregulation of Mvl contributed to the transition from nursing to foraging behavior. In our study, before day 63, the Mvl expression profile was similar in adult bees fed with all three diets but peaked on day 84 in adult bees fed with polyfloral diets. This may suggest that by the end of the experiment those bees fed with polyfloral diets performed significantly more outdoor feeding and foraging activities compared to their counterparts in the control groups but not compared to bees in the monofloral group. Because our data indicated low levels of food reserves (honey) in adult bees fed with polyfloral diets, the upregulation of *Malvolio* may suggest that the bees were increasing foraging in response to low food reserves (0.01 ± 0.00) (Table 2). Our data may follow findings on the gene expression profile of Seeley [61] and Page et al. [62] that indicated that expression was low when bees were focused on in-hive tasks (at the start of the experiments), but expression peaked when these bees performed outdoor activities (foraging) [63] and then decreased again when outdoor activities reduced.

### 4.6. Maltase

Adult bees fed with monofloral diets significantly upregulated the expression of *Maltase* compared to their counterparts fed with pollen patties and sugar syrup 21 days after the experiments were initiated. The upregulation of *Maltase* in these bees continued to the end of the 84-day experimental period. However, the differential gene expression of *Maltase* in adult bees fed with monofloral diets was similar to that of their counterparts fed with polyfloral diets throughout the experimental run. Furthermore, adult bees in control groups displayed similar *Maltase* expressions with their counterparts in polyfloral groups except at the end of the experiments on day 84, and, thus, differences were primarily between the control and monofloral diet groups. Our data indicated that adult bees fed with monofloral diets have relatively higher foraging behavior and may collect more pollen and nectar/honey for their colonies than their counterparts fed with pollen patties and sugar syrup. Our personal observations revealed that although control diets received the highest number of visits (95 per 15 min, compared to 8 visits for the same amount of time on monofloral diets), the average visit duration was longer (about 6 s) and the reward (pollen/nectar) was larger (pollen basket) than that of the other two diets. We also found that the pollen was used immediately for consumption and not for storage. *Maltase* is involved in energy metabolism as it converts maltose into glucose [64]. Glucose is the main energetic substrate for bee tissues, and it is stored in a form of glycogen that is used when energy demands are high [65,66,67].

### 4.7. Single-Minded Homologue 2

Gene expression levels of *Single-minded homolog* 2 (Sim 2) were significantly higher 21-day post treatments in adult bees fed with control diets (pollen and sugar syrup) compared to their counterparts fed with monofloral (anise hyssop) diets. Adult bees fed with monofloral or polyfloral diets displayed similar levels of gene expression during the 84-day experimental period. The *Single-minded homolog* 2 (Sim 2) gene encodes a transcription factor that is a master regulator of neurogenesis and plays a critical role in the development of the neurons, glia, and other nonneuronal cells that lie along the midline of the embryonic CNS [68,69]. The gene is also involved in locomotory behavior [70] which supports our personal observation that adult bees housed in flight cages fed with control diets were hyperactive [as a result, the mortality was much higher (population declined by 86.2%), Table 2)]. However, Pielage et al. [70] indicated that Sim 2 was important not only to control locomotion, but also to correctly specify the formation of the central complex and to correctly pattern the genital discs, as well as the anal pad anlage.

## 5. Conclusions

The availability of food (nutrients) is an important factor for the growth and survival of an organism; thus, malnutrition can impair immune function and consequently affect animal health, resistance to diseases, and survival [7]. In the case of honeybees, pollen and nectar are essential dietary sources and are vital for individual honeybee health and colony growth and development [7,20]. We found that the companion crops (monofloral and polyfloral) provided higher nutritional benefits to enhance honeybee health than the pollen patties and sugar syrup currently used by beekeepers. Furthermore, it has been reported that while single-species pollen has specific benefits, bees require pollen from diverse sources to maintain a healthy physiology and colony [25]. Our data on nuclear colonies indicated that a single-species diet (such as anise hyssop) is nutritionally adequate, as it is even much better than or comparable to polyfloral diets. The expression profiles of the selected genes in this study were significantly affected by diets (pollen and sugar patties, monofloral, and polyfloral) at the end of the experimental period. The potential relationships between diet and gene expression varied among these genes and was the highest with *Vitellogenin* and may translate into the behavior of adult bees. Based on the gene expression profile, the monofloral (anise hyssop) diets provided quality nutrients better than or comparable to those of polyfloral diets that were reported to be preferable to pollinators [7]. Additionally, we found that the companion crops provided higher nutritional benefits to enhance honeybee health than the pollen patties and sugar syrup currently used by beekeepers. It is critically important to provide honeybees with sustainable and nutritious food sources (plants, such as hyssop) throughout the season to enhance honeybee health. These companion crops could be integrated into the fabric of any apiary, as not only will they provide additional food sources (pollen and nectar) during the honey flow, but they also serve as good quality food resources during the off season (the absence of honey flow). These perennial crops (companion plants) could cover about 10% of the apiary and be planted as perimeter cropping, intercropping, mosaic cropping, and/or random packets, as well as part of the landscape of the apiary. Anise hyssop (perennial plant) could be one of the best choices as it provides nutritional supplements to enhance honeybee health.

## Figures and Tables

**Table 1:** Primers used for quantitative PCR of honeybee immune-linked genes for nutrition.

Gene	NCBI	Forward Primer (5′ to 3′)	Reverse Primer (5′ to 3′)	Function
*Cactus*	FJ546095.1	ACATAGTTCGG GCCACACTG	AGGTGCGGTTGCA GTATTCA	Immune response [35]
Immune deficiency (*IMD*)	NM_001163717.2	ACCCGCCAAAT GCCAATAGA	AGTCGATGGTGGTA ATGGTACT	Antimicrobial defense [37]
*Spaetzle*	XM_026444398.1	GTGTCAGTGGC GGTGACTAA	GCACGTGTTGATG TATCCGC	Member of the TOLL-immune signaling pathway against bacteria and fungi [36]
*Vitellogenin* (Vg)	NM_001011578.1	AACGCTTTTACTG TTCGCGG	TATGCACGTCCGAC AGATCG	Immune function and longevity [36]
*Maltase*	XM_006564751.3	CGAAAGCAGCAAC GAATGGG	ACAGGTTTATCGC TGTTACCGA	Energy metabolism [36]
*Malvolio* (mvl)	XM_006563052.3	TCCCCGCCAAGAT CACATTT	ACCACACCAAGTCT TGCACT	Involved in sucrose responsiveness [36]
*Single*-*minded homolog 2*	XM_016914389.2	TGCGATCGGGAGA AAGTGTC	TTTCGCCTCCAACT ACCGAC	Locomotor behavior [36]

**Table 2:** Mite infestations and colony development of adult bees fed with control (pollen patties and sugar syrup), monofloral (anise hyssop), and polyfloral (companion crops). Means in rows are not significantly different if followed by the same upper-case letter (*p* > 0.05). Means in columns are not significantly different if followed by the same lower-case letter (*p* < 0.05; Tukey test).

Treatments	^1^ Number of Mites per Hundred Adult Bees (Mean ± SE)		^2^ Colony Strength Parameters (Mean ± SE)			
			Bees		Honey	
	Before	After	Before	After	Before	After
Control	0.11 ± 0.01 aA	0.94 ± 0.61 aA	5.20 ± 0.52 aA	0.72 ± 0.67 aB	2.28 ± 0.30 aA	1.67 ± 1.07 aA
Monofloral	0.35 ± 0.14 aA	0.41 ± 0.19 aA	5.04 ± 1.00 aA	2.92 ± 0.82 aA	3.08 ± 0.32 aA	0.32 ± 0.19 aB
Polyfloral	0.01 ± 0.00 aA	0.20 ± 0.12 aA	5.54 ± 0.27 aA	1.12 ± 0.73 aB	2.14 ± 0.27 aA	0.01 ± 0.00 aB

^1^ The initial numbers of mites per hundred adult bees (mite infestations) were not significantly different among treatments. ^2^ Estimates were made in tenths of standard Langstroth frames [33,34].

## Data Availability

The original contributions presented in this study are included in the article. Further inquiries can be directed to the corresponding author.
